# Low follistatin level is a causal risk factor for spontaneous abortion: a two-sample mendelian randomization study

**DOI:** 10.3389/fendo.2023.1255591

**Published:** 2024-01-03

**Authors:** Chen Gong, Wenzhi Yang, Xue Liu, Xinliang Li, Yutong Wang, Chan Tian

**Affiliations:** ^1^ State Key Laboratory of Female Fertility Promotion, Center for Reproductive Medicine, Department of Obstetrics and Gynecology, Peking University Third Hospital, Beijing, China; ^2^ National Clinical Research Center for Obstetrics and Gynecology, Peking University Third Hospital, Beijing, China; ^3^ Key Laboratory of Assisted Reproduction, Peking University, Ministry of Education, Beijing, China; ^4^ Beijing Key Laboratory of Reproductive Endocrinology and Assisted Reproductive Technology, Beijing, China; ^5^ Department of Neurology, Peking University Third Hospital, Beijing, China; ^6^ Department of Medical Genetics, Center for Medical Genetics, Peking University Health Science Center, Beijing, China

**Keywords:** follistatin, spontaneous abortion, mendelian randomization, abortion, recurrent pregnancy loss

## Abstract

**Background:**

Recurrent pregnancy loss is a distressing event during pregnancy, and understanding its causal factors is crucial. Follistatin, a glycoprotein involved in folliculogenesis and embryogenesis, has been implicated as a potential contributor to the risk of spontaneous abortion. However, establishing a causal relationship requires rigorous investigation using robust methods.

**Methods:**

In this study, we utilized mendelian randomization (MR), a powerful genetic epidemiological approach, to examine the causal relationship between follistatin levels and spontaneous abortion. We obtained instrumental variables strongly associated with follistatin levels from large-scale genome-wide association from the IEU database. The inverse variance weighting (IVW) method was taken as gold standard. We also performed sensitivity test to evaluate the robustness of our result.

**Results:**

MR analysis revealed a significant causal relationship between low follistatin levels and spontaneous abortion (p = 0.03). Sensitivity analyses, including pleiotropy test, heterogeneity test, and leave-one-out analysis, all supported the robustness of our findings.

**Conclusion:**

Our study provides compelling evidence supporting the causal relationship between low follistatin levels and increased risk of spontaneous abortion. These findings underscore the importance of follistatin in the etiology of spontaneous abortion and suggest potential preventive interventions. Modulating follistatin levels or relevant pathways could hold promise for reducing the incidence of spontaneous abortion and improving reproductive outcomes. The utilization of MRs strengthens the validity of our results by mitigating confounding and reverse causality biases. Further research is needed to elucidate the underlying molecular mechanisms and explore therapeutic strategies targeting follistatin levels.

## 1 Introduction

Spontaneous abortion is a frequently encountered complication during pregnancy, characterized by the loss of pregnancy before 20 weeks of gestation (1). Approximately 9-20% of all recognized pregnancies result in spontaneous abortion. Among these, 3-5% of couples face the challenge of two or more clinically recognized pregnancies ending in failure, known as recurrent pregnancy loss (RPL) (2, 3). RPL can be devastating, bringing great trauma to both the patient and their family (4). Various factors contribute to RPL, with chromosomal abnormality being the most common, responsible for over half of RPL cases (5). Additionally, 10%–15% of women with multiple pregnancy losses exhibit uterine anomalies, such as partial or complete septum (6, 7). Hormonal causes such as luteal phase defect, pregestational diabetes mellitus, and polycystic ovary disease can lead to RPL (8). Moreover, immune disorders may contribute to RPL by dysregulating trophoblast function and endometrial angiogenesis (9). For example, primary antiphospholipid syndrome (APS), present in one-third of RPL patients, is associated with elevated serum anti-phospholipid antibody (aPL) levels. Increased aPL levels reduce placental hormone production, impair trophoblast function, and result in pregnancy loss and other obstetric complications (10). Women with celiac disease often have elevated anti-transglutaminase type 2 (anti-TG2) autoantibodies, leading to reduced blood vessel formation and disrupted endometrial angiogenesis, contributing to RPL (11). Infections, exposure to environmental agents, and elevated homocysteine levels are also implicated in RPL (12). Nevertheless, the understanding of RPL remains significantly limited, as almost 50% of RPL cases are still categorized as unexplained (9).

Follistatin (FST) is a secreted protein that primarily synthesized and secreted by the liver, mainly implicated in suppressing follicle-stimulating hormone (FSH) activity through autocrine or paracrine mechanisms (13–15). Notably, FST serves as a binding protein and regulator in the transforming growth factor-beta (TGF-β) signaling pathway, selectively binding to ligands such as activins and bone morphogenetic proteins (BMP) (13, 16, 17). It restrained granulosa cell proliferation and steroidogenesis by neutralizing the action of activin (18–20). It also enhanced basal estradiol and progesterone production (21, 22), promoting cell invasion *via* the ALK4-SMAD2/3-SMAD4 signaling pathway (23–26).

Serum FST increased significantly throughout gestation until the first day of parturition and declined afterward (27). Evidence showed its possible role as chemokine to induce trophoblast migration and invasion through the enhanced JNK signaling, contributing to maintain trophoblast function and promote placental development (28, 29). FST was upregulated in the decidua during early pregnancy, and women with RPL were observed to have a lower endometrial expression of FST during the luteal phase (30). A lower FST level in endometrium stromal cells of women with RPL was also observed (30). Conditional knockout of mice uterine *Fst* can cause severe fertility defects, reduced responsiveness to estrogen and progesterone signals, impaired artificial decidualization, and an unreceptive environment for embryo attachment. These findings suggest that *Fst* may play a crucial role in facilitating uterine receptivity (31). A decreased FST level was also observed in serum and placenta of women with preeclampsia (PE) (32–34), resulting in impaired trophoblast function through upregulating GDF11 levels in trophoblasts. The dysregulation of the FST-GDF11-Smad2/3 axis may be critical to trophoblast function, which adds more evidence to the essential role of FST on trophoblast during pregnancy (35).

Mendelian randomization (MR) is an epidemiological tool based on genetic variants related to exposure factors, helping to assess the association of these gene variations with outcomes such as disease onset or mortality. Its core relies on using genetic data as a bridge to investigate causal relationships between a particular exposure and a specific outcome (36, 37). Randomized controlled trials (RCTs) have long been recognized as the gold standard for causal inference, yet it is costly and complicated to conduct. Similar to RCTs that randomly assign participants to a trial or control group, MR studies “randomize” participants based on one or more gene alleles that influence risk factors and attempt to determine if carriers of these genetic variations have different disease onset risks compared with non-carriers (38). Traditional observational study designs rely on exposure obtained through questionnaires, biochemical assays, or imaging, whereas genetic variations exist at birth and remain stable throughout life. Notably, information of genetic variation and diseases is easy to acquire through open-dataset, and since it leaves out the complicated implementation process and ethical restriction, it is much easier to conduct compared with RCTs (39). In view of these incomparable advantages of MR, here we performed a two-sample MR analysis of the GWAS summary data from the UK Biobank and EBI database so as to find whether there is a potential causal association between FST level and spontaneous abortion, trying to provide novel evidence in this field of research.

## 2 Materials and methods

### 2.1 Study design

**Figure 1:**
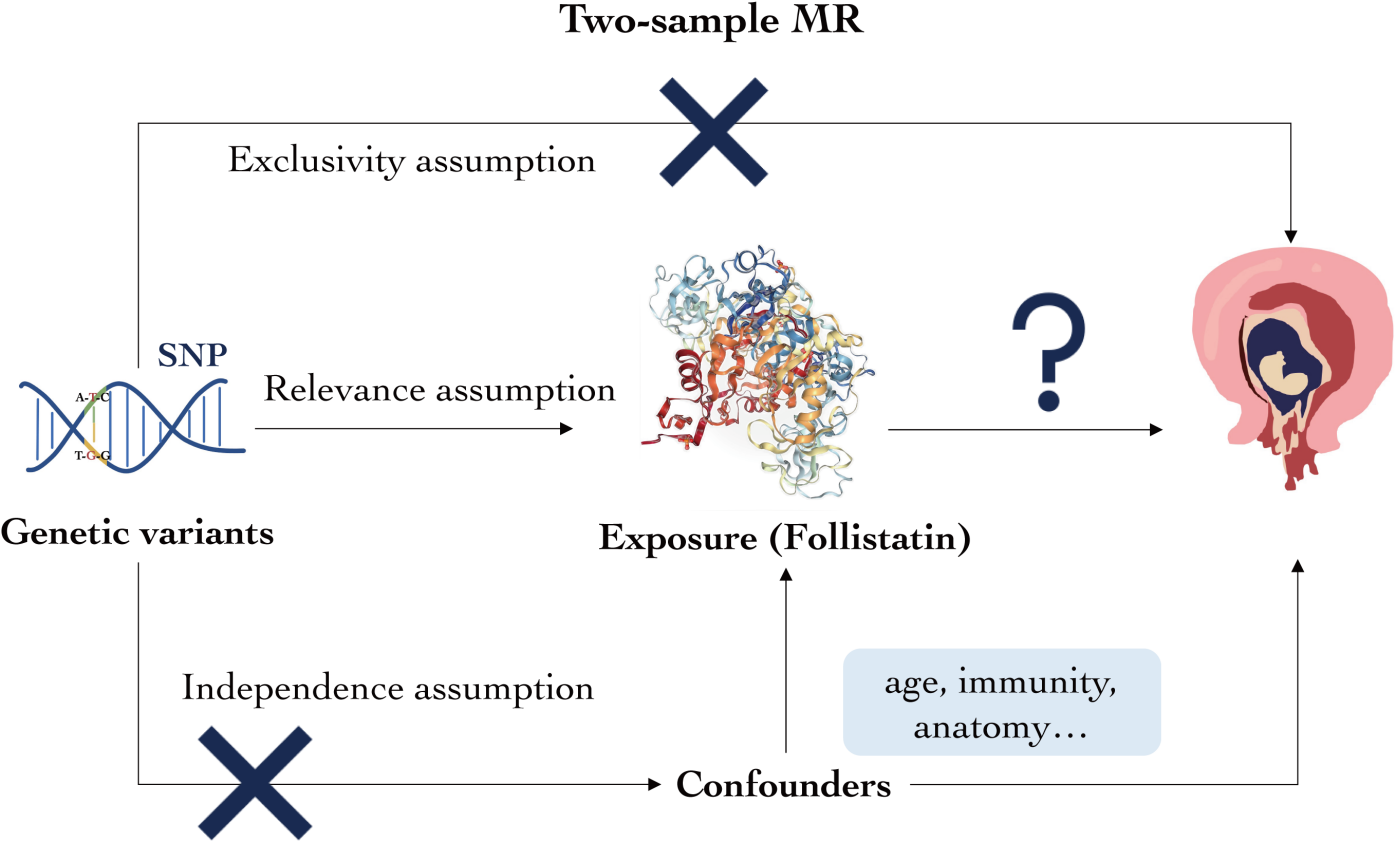
Overview of the study’s key factors. Genetic variant, exposure, and outcomes are as described. Following the three assumptions, genetic variant must be closely correlated with the exposure but cannot be associated with neither the confounders nor the outcomes.

Here, we conducted a two-sample MR analysis to examine the possible causal association between FST level and spontaneous abortion. **Figure 1** provides an overview of the study’s key factors, including the exposures, outcomes, and genetic instruments. The study was based on previously published materials and public databases and received ethical approval and participant consent from the relevant institutional review committees.

The genetic variants in this study are fully considered based on the three principles below throughout our analysis. First is relevance assumption: the genetic variant must be closely correlated with follistatin levels; second is independence assumption: the genetic variant cannot be associated with any possible confounders of follistatin levels or spontaneous abortion; and third is exclusivity assumption: the genetic variant cannot be related to the relevant outcomes.

### 2.2 Data sources

#### 2.2.1 Exposure population and data

We extracted exposure data from a previous study (40), downloaded from the website of IEU OPEN GWAS PROJECT (https://gwas.mrcieu.ac.uk/, GWAS ID: ebi-a-GCST90012080).

#### 2.2.2 Outcome population and data

We extracted outcome data from a UK Biobank study (Dataset ID: ukb-d-O03), downloaded from the website of the IEU OPEN GWAS PROJECT (https://gwas.mrcieu.ac.uk/).

### 2.3 Statistical analysis

All the data processing and statistical analyze are performed using the R4.2.2 software (Lucent Technologies, USA). The MR, heterogeneity test and pleiotropy test were carried by the “Two Sample MR” package (41).

### 2.4 Extraction of instrumental variables

We performed two-step filtering to eliminate those SNPs that do not satisfy the relevance assumption and obtained the satisfying instrumental variables (IVs). We extracted SNPs that are (1) closely related to the exposure (follistatin level) at a genome-wide significance threshold of *p* < 5 × 10^−8^. 2) without linkage disequilibrium (LD) (linkage disequilibrium r^2^ < 0.05), since we should make sure that no correlation LD between selected IVs and potential confounding factors exist. The R^2^ value was calculated for these IVs to assess their association with the exposure (42).

### 2.5 Elimination of confounding factors

We used PhenoScanner (Version 2), a database of human genotype–phenotype associations to figure out whether the confounding factors such as anatomical abnormalities, hormonal imbalances (PCOS, luteal phase defect, etc.), and immune disorders (antiphospholipid syndrome, lupus erythematosus, etc.) may influence our result according to the independent assumption (43, 44). Over 65 billion associations and over 150 million unique genetic variants are recorded in PhenoScanner. We applied the “Phenoscanner” R package to investigated each of the five IVs and their corresponding phenotypes (43, 44). Any IVs exhibiting associations with confounding factors were excluded from the analysis, applying stringent criteria (*p* < 1 × 10^−5^, r^2^ > 0.8).

### 2.6 Sensitivity test

To make sure that our MR results are robust enough for us to come up with a causal conclusion, we extensively performed three aspects of sensitivity test, namely, heterogeneity test, pleiotropy test, and leave-one-out analysis test. R software was used to visualize results with depicting scatter plot, forest plot, etc.

### 2.7 Two-sample MR analysis

In two-sample MR analysis, five methods are commonly used: MR-Egger, Weighted Median, Inverse Variance Weighted, Simple Mode, and Weighted Mode. Among these methods, Inverse Variance Weighted is widely accepted and considered the most effective, as it accounts for heterogeneity when assessing causality. However, the other four methods also demonstrate robustness to varying degrees. MR-Egger is particularly useful when there is potential violation of instrumental variable assumptions, such as pleiotropy. It estimates the causal effect while allowing for average pleiotropic bias (45). The Weighted Median method provides a robust estimate by considering the median of all possible instrumental variable estimates, even when up to 50% of the instruments are invalid. This method is advantageous in situations where some instrumental variables may be biased or weak. The Simple Mode and Weighted Mode methods combine the estimates from multiple instruments by either taking the mode or using weighted averages. To evaluate the causal risk of FST levels, we performed all five methods. A positive result from any one or more of these methods would indicate a potential causal risk associated with FST levels.

## 3 Results

### 3.1 Study population

The exposure population pertains to follistatin levels, sourced from 39 cohorts of European ancestry. These data were thoroughly cleaned and summarized in a previously published study in 2020 (40), where a genome-wide meta-analysis of 90 cardiovascular-related proteins across 15 studies were identified. We extracted identified genetic variants related to follistatin levels from the recorded ebi-a dataset of IEU open GWAS project (GWAS ID: ebi-a-GCST90012080). In this dataset, 21,758 samples are involved, with 13,022,208 SNPs being reported (40). All of them are of European ancestry. The effect allele (EA), other allele (OA), beta coefficients, p value, and standard error (SE) are also included in this dataset for further investigation.

For the outcomes, summary-level data were obtained from the UK Biobank study (46). In the UK Biobank, pregnancy loss was defined as a history of self-reported spontaneous abortion or termination. We utilized the second round of Neale Lab’s genome-wide association analyses in the UK Biobank, obtaining 1,150 female patients with spontaneous abortion and 360,044 matched controls of European ancestry and 9,543,298 detected SNPs.

### 3.2 Five SNPs are validated as instrumental variables

MR relies on the idea of random allocation of genetic traits. If the frequency of SNPs harmonizes with the alteration of the exposure, we can tentatively deduce that the SNP is correlated with the exposure. We screen the total of 13,022,208 SNPs in the exposure dataset based on the relevance assumption and independence assumption mentioned in the “study design” and finally achieved five SNPs that satisfied for IVs. We presented some detailed information for these SNPs such as effect allele frequency, standard error, and effect allele (**Table 1**). In addition, we calculated the R^2^ value for the IVs, which help to explain the extent of exposure (**Table 2**).

**Table 1:** General data for five SNPs as instrumental variables.

SNP for IV					Miscarriage (outcome)				Follistatin (exposure)				
Chr	Position	SNP ID	EA	OA	Beta	EAF	SE	*p* value	Beta	EAF	SE	*p* value	R^2^
9	92228559	rs10908903	G	T	-1.43E-04	0.4677	0.0106	0.2850	0.0603	0.4587	0.0109	2.76E-08	3.43E-03
2	27730940	rs1260326	C	T	1.05E-04	0.6069	0.0107	0.4381	-0.1323	0.6024	0.0106	9.58E-36	3.21E-03
15	43726625	rs150844304	C	A	-5.26E-04	0.0245	0.0109	0.2187	0.2466	0.0294	0.0321	1.46E-14	6.98E-04
5	53327571	rs31226	C	T	2.62E-04	0.6064	0.0123	0.0541	-0.1289	0.5979	0.0107	1.56E-33	7.87E-04
12	57791833	rs7974833	C	T	9.15E-05	0.2367	0.0321	0.5561	0.0849	0.2350	0.0123	5.61E-12	1.55E-04

Chr, chromosome; SNP, single-nucleotide polymorphism; EA, effect allele; OA, other allele; EAF, effect allele frequency; SE, standard error.

**Table 2:** Evaluation of instrumental variables.

SNPs for IV		Follistatin level (exposure)			
Chr	Position	SNP ID	MAF	beta	SE
9	92228559	rs10908903	0.4587	0.0603	0.0109
2	27730940	rs1260326	0.6024	−0.1323	0.0106
15	43726625	rs150844304	0.0294	0.2466	0.0321
5	53327571	rs31226	0.5979	−0.1289	0.0107
12	57791833	rs7974833	0.235	0.0849	0.0123

IV, instrumental variable; Chr, chromosome; SNP, single nucleotide polymorphism; MAF, minor allele frequency; Beta, the effect size; R^2^, IV explains the extent of exposure.

### 3.3 MR analysis showed that follistatin level is a causal risk for spontaneous abortion

**Figure 2:**
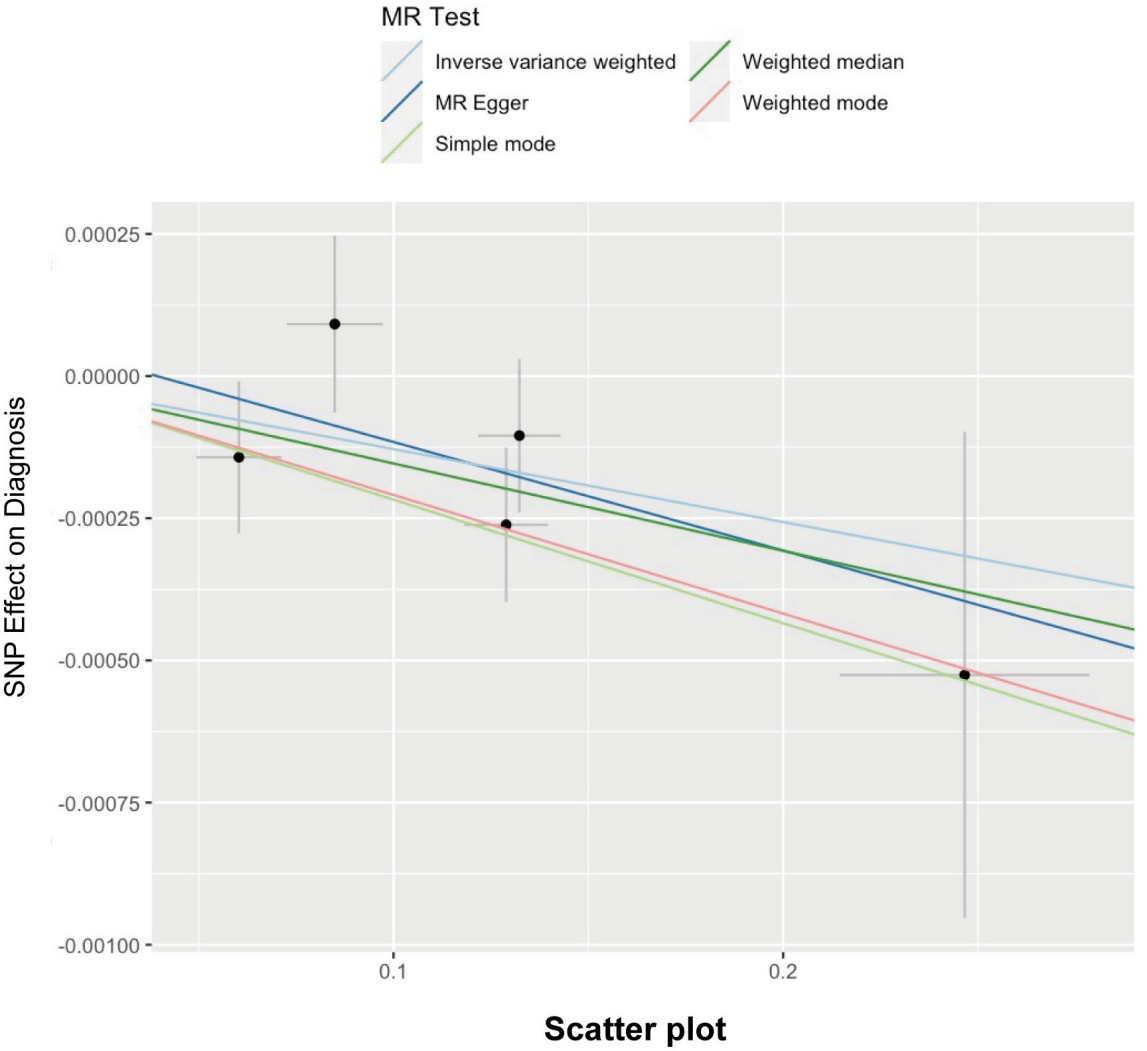
Scatter plot illustrating the distribution of individual ratio estimates of follistatin levels with spontaneous abortion as the outcome. Trend lines derived from five different 2SMR methods are also included in each scatter plot to indicate cause and effect.

Here, we adopted five methods to evaluate the follistatin level effect on the risk of spontaneous abortion, and the results are shown in **Table 3** and **Figure 2**. Considering the absence of neither heterogeneity nor pleiotropy (which we would describe in detail on the next part), we selected IVW as the main method as well as the gold standard for determining the causal effect of FST level on the risk of spontaneous abortion (47). We found that the IVW method showed a *p* value of 0.03517563 (<0.05) and *b* of −0.001282787 (<0), indicating the causal relationship between low FST level in the European population.

**Table 3:** Causal effect between follistatin level and spontaneous abortion by different MR analysis methods.

Exposure	Outcome	Method	nSNP	*p* value	beta	R^2^
Follistatin levels	Spontaneous abortion	MR–Egger	5	0.36562849	-0.001908907	3.43E-03
Follistatin levels	Spontaneous abortion	Weighted median	5	0.03961415	-0.001535967	3.21E-03
Follistatin levels	Spontaneous abortion	Inverse variance weighted	5	0.03517563	-0.001282787	6.98E-04
Follistatin levels	Spontaneous abortion	Simple mode	5	0.09652113	-0.002171829	7.87E-04
Follistatin levels	Spontaneous abortion	Weighted mode	5	0.10618687	-0.002087265	1.55E-04

### 3.4 Sensitivity analysis

In this work, sensitivity analysis is performed to (1) evaluate whether the results are robust and the conclusions are reliable; (2) assess whether the results have potential biases (such as genetic pleiotropy and data heterogeneity); and (3) evaluate whether there is a certain instrumental variable that significantly affects the outcome variable.

**Figure 3:**
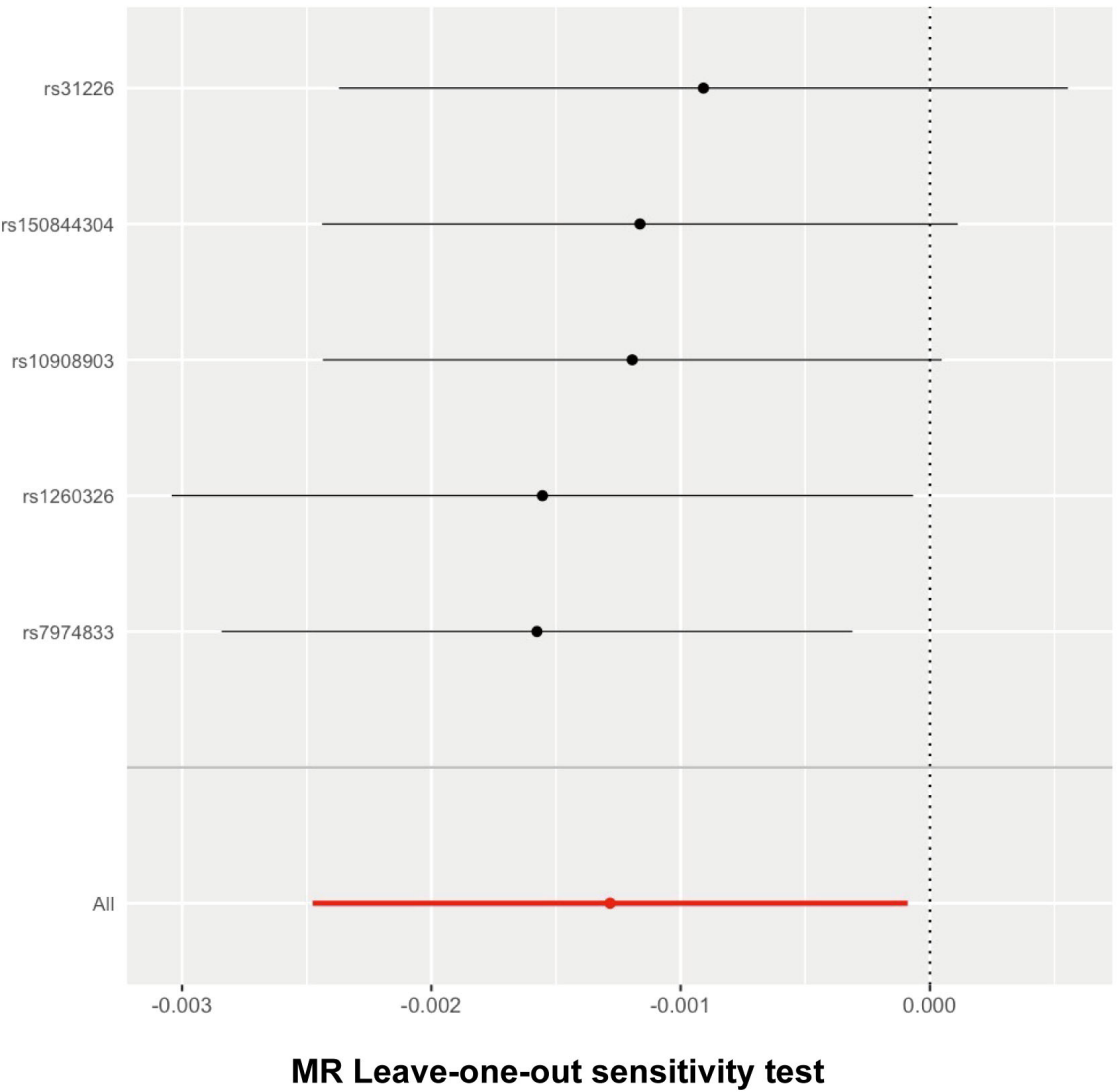
Leave-one-out analysis for follistatin levels on spontaneous abortion. The given dark dots indicate effect measures from IVW-MR analysis excluding specific SNPs. Red line indicates pooled analysis including all SNPs by the IVW-MR method (plotted for comparison).

We detected no heterogeneity in the five IVs that we chose for the spontaneous abortion (MR–Egger Q statistics = 2.738052; Qdf = 3; Qpval = 0.4337995; IVW Q statistics = 2.875518; Qdf = 4; Qpval = 0.5788685) (**Table 4**). We then focused on the pleiotropy using the MR–Egger method. The intercept with the Y-axis represents the horizontal pleiotropy. Zero horizontal pleiotropy is one of the prerequisites of applying the MR method according to the exclusive assumption. Here, we noticed no horizontal pleiotropy existed in our MR analysis results (Egger_intercept = 7.505441e-05; se = 0.0002024312; *p* value = 0.7354456) (**Table 5**). In the “leave-one-out” analysis, we sequentially removed each SNP and calculated the MR effect of the remaining SNPs. We noticed that the removal of any of these individual SNPs did not result in significant changes of the overall causal estimation effect (**Figure 3**). Taken together, these results suggest that our findings were robust and the exception of single IV exert no difference on the overall estimated causal effect.

**Table 4:** Heterogeneity statistics.

Method	Q Q_df	Q_pval
MR–Egger	2.738052	0.4337995
Inverse variance weighted	2.875518	0.5788685

**Table 5:** Pleiotropy statistics of MR analysis.

Method	Egger regression of intercept	SE	*p* value
MR–Egger	7.51E-05	0.000202431	0.7354456

**Figure 4:**
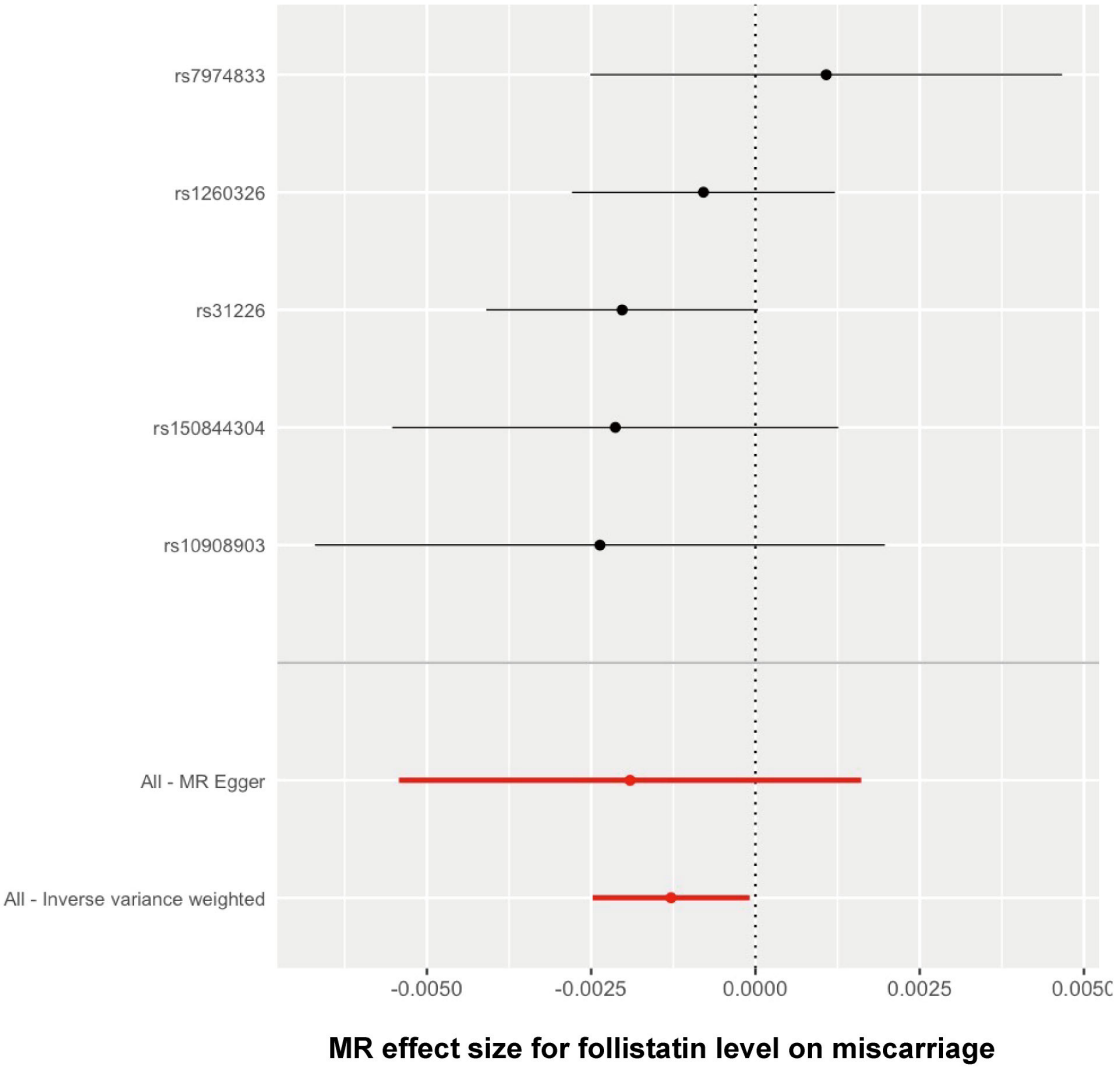
Forest plot showing the causal effect of each single SNP on the risk of miscarriage.

The Wald ratio method was used to estimate the causal effect of each individual SNP on the risk of spontaneous abortion. The findings have been presented in a forest plot to provide a visual representation (**Figure 4**). The threshold of significance for the forest plot remained controversial. It can be defined as either *p* < 0.05 or *p* < 0.05/n (n refers to the number of SNPs). Here, comprehensively regarding the *p* values for each single SNP on the outcome (**Table 1**), the leave-one-out analysis test (**Figure 3**), and all the SNPs combined (**Figure 4**), it was quite clear that a causal association existed between follistatin level and the risk of spontaneous abortion.

## 4 Discussion

In this study, we employed two-sample MR to investigate the causal relationship between FST levels and spontaneous abortion. The evidence from MR analysis indicates that low follistatin level was a causal risk factor for spontaneous abortion and these results were generally reliable as the sensitivity analysis strongly supports. These findings have important implications for understanding the pathogenesis of spontaneous abortion and may contribute to the development of potential preventive and therapeutic strategies.

It is widely acknowledged that establishing proper placentation involves sequential processes, notably trophoblast invasion and angiogenesis (48). The orchestrated interplay of angiogenic processes and steroid hormones induces transformative changes in the endometrium, facilitating its receptivity to the blastocyst and initiating the implantation process (49). Successful placentation and the commencement of pregnancy hinge on prerequisites such as endometrial angiogenesis, decidualization, and trophoblast invasion (48, 50, 51). Any dysregulation during the above processes may lead to pregnancy failure and RPL. High levels of anti-annexin V antibody can bind to trophoblast cells, affecting trophoblast invasiveness and causing defective placentation (52). Also, lots of proteins and related pathways have been recognized to be involved in regulating trophoblast function, such as TGF-β which governs the differentiation program of extravillous trophoblasts in the developing human placenta (53).

The underlying biological mechanisms linking low follistatin levels with spontaneous abortion warrant further investigation. Follistatin not only has an inhibitory effect on FSH secretion from cultured anterior pituitary cells (54) but also is involved in trophoblast invasion and embryonic development. FST acts as an antagonist to the TGF-β superfamily and thereby modulates important signaling pathways such as JNK signaling, ALK4-SMAD2/3-SMAD4 signaling, and FST-GDF11-Smad2/3, further affecting trophoblast function (55). It is hypothesized that reduced follistatin levels may disrupt multiple pathways such as JNK signaling, ALK4-SMAD2/3-SMAD4 signaling, and FST-GDF11-Smad2/3, leading to trophoblast dysfunction and causing impaired implantation and defective placental development, ultimately resulting in abortion. Future research should focus on elucidating the specific molecular mechanisms through which follistatin influences pregnancy outcomes.

Our results are consistent with previous studies that have reported a probable association between low follistatin levels and adverse pregnancy outcomes (30, 56–58). For instance, Prakash et al. (30) observed a dramatic decrease of FST expression in endometrial stromal cells of women with spontaneous abortion. However, it should be noted that some studies reported no significant decrease of follistatin in the serum of women with spontaneous abortion (59, 60). These discrepancies may arise from variations in sample size and characteristics, since it only includes around 10 abortion samples and 10 control samples.

Although previous studies have pointed out a possible association between FST and spontaneous abortion, there is no absolute evidence on a genetic aspect. Establishing a direct causal link between follistatin and spontaneous abortion presents challenges due to confounding effects from factors such as lifestyle and environmental influences. To overcome this challenge, a promising approach is the utilization of MR, which leverages genetic variants as IVs to infer causal relationships. In our study, we utilized MR to present novel evidence that demonstrates a causal relationship between low FST levels and spontaneous abortion. This finding holds significant implications for the field of reproductive health. Our results suggest that interventions targeting increased FST levels may have the potential to reduce the incidence of spontaneous abortion, which is a devastating outcome for numerous couples. However, despite the valuable insights gained from this study, several limitations should be acknowledged. We recognize that our analysis only included the European population, introducing the possibility of potential bias associated with differing ancestries.

Our study yields meaningful clinical implications. First, it indicates that FST levels might be integrated into routine antenatal assessments to evaluate the risk of pregnancy failure. This is particularly crucial for individuals with a history of spontaneous abortion, where assessing their FST levels may serve as a predictive indicator for the occurrence of RPL. Concurrently, FST may function as a prospective biomarker for targeted interventions like hormonal therapies or lifestyle adjustments and the development of personalized medical strategies. Future studies could explore interventions aimed at modulating follistatin levels to potentially prevent or mitigate the risk of spontaneous abortion. Moreover, investigations into the long-term effects of low follistatin levels on maternal and offspring health outcomes would be valuable for a comprehensive understanding of the implications.

In conclusion, our study contributes to the growing body of reliable evidence supporting the critical role of FST in successful pregnancy outcomes and highlights it as a promising therapeutic target. We remain hopeful that further research, conducted with larger sample sizes based on our observations, will provide additional insights into the underlying mechanisms that link FST and pregnancy outcomes.

## Data availability statement

This study analyzed the exposure data acquired from EBI database and the outcome data sourced from the UK Biobank. Both datasets are publicly accessible and can be found on the IEU Open GWAS Project website (https://gwas.mrcieu.ac.uk/).

## Author contributions

CG: Formal analysis, Investigation, Methodology, Writing – original draft, Writing – review & editing. WY: Methodology, Writing – review & editing. XL: Writing – review & editing. XLL: Writing – review & editing. YW: Validation, Writing – review & editing. CT: Conceptualization, Methodology, Supervision, Writing – review & editing.
